# Beyond advertising: New infrastructures for publishing integrated research objects

**DOI:** 10.1371/journal.pcbi.1009651

**Published:** 2022-01-06

**Authors:** Elizabeth DuPre, Chris Holdgraf, Agah Karakuzu, Loïc Tetrel, Pierre Bellec, Nikola Stikov, Jean-Baptiste Poline

**Affiliations:** 1 NeuroDataScience–ORIGAMI Laboratory, McGill University, Montreal, Quebec, Canada; 2 The International Interactive Computing Collaboration (2i2c), Berkeley, California, United States of America; 3 International Computer Science Institute, Berkeley, California, United States of America; 4 NeuroPoly Lab, Polytechnique Montreal, Montreal, Quebec, Canada; 5 Montreal Heart Institute, Montreal, Quebec, Canada; 6 Centre de recherche de l’Institut universitaire de gériatrie de Montréal, Montreal, Quebec, Canada; 7 Department of Psychology, Université de Montréal, Montreal, Quebec, Canada; Johns Hopkins University, UNITED STATES

Moving beyond static text and illustrations is a central challenge for scientific publishing in the 21st century. As early as 1995, Donoho and Buckheit paraphrased John Claerbout that “an article about [a] computational result is advertising, not scholarship. The actual scholarship is the full software environment, code and data, that produced the result” [1]. Awareness of this problem has only grown over the last 25 years; nonetheless, scientific publishing infrastructures remain remarkably resistant to change [2]. Even as these infrastructures have largely stagnated, the internet has ushered in a transition “from the wet lab to the web lab” [3]. New expectations have emerged in this shift, but these expectations must play against the reality of currently available infrastructures and associated sociological pressures. Here, we compare current scientific publishing norms against those associated with online content more broadly, and we argue that meeting the “Claerbout challenge” of providing the full software environment, code, and data supporting a scientific result will require open infrastructure development to create environments for authoring, reviewing, and accessing interactive research objects.

## Publishing as curating, promoting, and archiving content

Scientific publishing platforms—traditionally, scientific journals—fulfill a variety of roles in their communities. Three of the most prominent of these are curating, promoting, and archiving research. Although these roles have adapted to online spaces, they have not been fundamentally reshaped. Indeed, contemporary scientific articles are disseminated primarily as portable document formats (PDFs), directly translating paper-based workflows into digital workspaces. Here, we briefly review how publishing fulfills these roles today: curation via peer review, short-term promotion via online dissemination, and long-term access via archiving.

Across many kinds of media, curating online content is challenging both due to its scale and its style of interaction, which often blurs the boundary between creating and consuming information. For scientific publishing, formal and independent peer review is widely considered to be a key demarcation [4] and provides an immediate mechanism to curate research objects. Curation in peer review involves checks on a submission’s ethical and scientific rigor, in addition to its relevance to a particular research community. Even as many other forms of curation are possible—including crowd sourced or algorithmically driven [5]—these remain relatively uncommon in neuroscience (cf., arxiv-sanity.com).

In addition to curating (i.e., reviewing and selecting) research objects, publishing also serves an important role in promoting and archiving content. This occurs in the short term through activities such as website hosting and advertising on social media platforms [6]. Ongoing promotion to an ever-evolving scientific community is enabled through the long-term archiving and the references system. These roles can be fulfilled independently or in an arbitrary order. For example, online interactions have allowed peer review to expand into postpublication peer review on platforms such as PubPeer (https://pubpeer.com) and Sciety (https://sciety.org; [7]).

Even as scientific publishers have successfully moved online, they have not yet embraced the full potential of web-first workflows. We briefly review how 2 norms of online content, connectivity and interactivity, are currently reflected in scientific publishing before arguing for infrastructure that allows for more directly interactive and reusable content.

## Rich linking for research objects: Connecting through hybrid content types

Much of the rich, content-driven interactivity of the web depends on access to structured data such as user content on social media platforms. To separate out this content from its presentation, data formats such as XML have been developed to link online content with its supporting resources [8]. Although scientific publishing workflows are largely built around the XML format, the need to output PDF documents means that resources that cannot be directly embedded—such as executable code or supporting data—have been largely excluded from academic publishing. Thus, the scientific narrative has historically been detached from its other associated research objects.

Recently, growing awareness of this problem has led to an increase in publishing what we term “hybrid research objects.” Hybrid research objects are distinct from traditional publications in that they make multiple content types available in the same object; that is, they contain narrative text and at least one or more examples of code, data, and computation (e.g., [9]). Multiple paths exist to make these objects available. One path is to include direct links to each resource such as through data and code availability statements [10], without constraining their format or content. Alternatively, some publishers require that linked research objects adhere to specified standards and are explicitly included in the review process. For example, the journal *Scientific Data* from Nature Research publishes descriptors of datasets [11] that include links to dedicated, domain-relevant data hosting infrastructure such as OpenNeuro (https://openneuro.org). Importantly, this raises new questions on how to appropriately handle their peer review, questions for which there is no current consensus [12].

As hybrid research objects have become more prominent, best practices in publishing these objects continue to evolve. We hope to see more hybrid research objects where each linked object is formatted with domain-relevant standards (e.g., neuroimaging data organized according to a domain standard such as the Brain Imaging Data Structure (BIDS); [13]) and bidirectionally linked using persistent identifiers. Nonetheless, because the linked research objects are hosted on unique platforms without clear checks on interoperability across the hybrid object components, it can be difficult to interact with the code, data, or their combination, for example, when trying to perform minimal quality checks on a dataset. It further prevents eventual readers from assessing the reproducibility or generalizability [14] of presented results. Enhancing this experience requires making these research objects interoperable, improving their reusability. Here, we introduce the idea of “integrated” research objects to explicitly test the interaction of included research objects in reproducing a scientific result.

## Bridging the gaps: Interactive and integrated research objects

Interactivity is an attractive feature of online content and one that scientists have been especially eager to adopt [15]. This enthusiasm has spurred development of platforms such as Bokeh (https://bokeh.org) and Plotly (https://plotly.com), enabling scientists to provide multiple views of their data through interactive figures and dashboards. Although this work is impressive, it is limited: Researchers remain unable to modify or reexecute the code used to generate these figures when shared through HTML documents. This hinders deep engagement with the presented results.

Achieving deeper interactivity requires interaction between the code, data, and computation supporting a scientific result. One approach to achieve this is to focus on what we call “integrated research objects.” Integrated research objects not only make multiple kinds of research objects available and tightly coupled, but they also do so in formats (e.g., computational notebooks) that foreground their interaction by allowing reexecution. In doing so, they offer a clear answer to the Claerbout challenge.

There are limits on the kinds of experiments that can be supported through integrated research objects; for example, experiments relying on cell cultures or other biological samples may only have digital representations of the statistical analyses and end results rather than the experiments themselves. Nonetheless, researchers should be encouraged to provide access to research objects that can be digitized. This is particularly important for computational work, where experiments are carried out in silico and so computation and the resulting narrative are closely linked.

Despite their immediate appeal, the infrastructure required to support integrated research objects is less straightforward. In particular, authoring, curating, and archiving these research objects all introduce significant challenges. Further, requiring that these objects be archivable imposes strong constraints on the kinds of technologies that can be used. Most archival services discourage submitting complex HTML objects with external dependencies as these documents are unlikely to retain their full functionality with evolving versions of HTML, JavaScript, and web browsers [16].

To sidestep this concern, current pilots for publishing integrated research objects consider them as secondary to a traditional, archivable article. For example, *eLife* authors can develop additional, web-first materials to accompany their accepted research articles. Codeveloped with Stencila (https://stenci.la), these executable research articles (ERAs) inherit their structure from the Jupyter notebook [17] format. ERA development has explicitly focussed on improving the authoring experience, and authors are supported in ensuring that all relevant code and data files are included in the ERA environment. While this support reduces the technical barrier in creating integrated research objects, it also means that ERAs are necessarily only developed at the end of the publication process after scientific analyses are finalized. In this way, the traditional, narrative text–based document remains privileged as the primary research object.

Centering integrated research objects will require infrastructure development to both ease the authoring experience as well as represent these objects in an archivable format. Although several standards for integrated research objects could serve as potential starting points, we argue that sustainable development demands open standards with multistakeholder governance and leadership to ensure that resulting specifications are not driven by a single stakeholder.

## Authoring integrated research objects with open standards

Perhaps the 2 most broadly adopted standards for integrated research objects are the RMarkdown (https://rmarkdown.rstudio.com) and Jupyter notebook [17] formats. Both technologies allow researchers to create integrated research objects that include narrative text, code, and computation, although they do so using different internal implementations. Specifically, RMarkdown is based on YAML and markdown formats, while Jupyter notebook is based on the JSON format.

Recent development on Jupyter Book (https://jupyterbook.org) has led to the creation of a MyST markdown format (https://myst-parser.readthedocs.io) that extends Jupyter to build from a combination of YAML and markdown, improving handling for scientific publishing use cases. Thus, RMarkdown and MyST allow researchers to directly describe their scholarship—the code, data, and computation that support a given scientific result—such that it can be easily source controlled and archived. They each also enable generation of user-focused HTML and PDF documents, including PDFs formatted for several major scientific journals (using, e.g., “rticles”; [18]), from user-provided markdown content.

These technologies differ, however, in that RMarkdown development is controlled by a single stakeholder, RStudio. Although its product is openly licensed, developed with community consultation, and freely available, decision-making power rests with RStudio employees. This model is distinct from multistakeholder governance, in which formats are not controlled by individual entities but instead benefit from consensus across organizations. We thus focus on standards developed within the Jupyter ecosystem.

Open standards development within Jupyter has enabled other initiatives such as Stencila and Curvenote (https://curvenote.com) to overlay with additional views and functionality. Integrating these technologies into existing standards (e.g., the Journal Article Tag Suite (JATS) XML format) via translation or conversion processes remains an active area of work. Perhaps their largest departure from existing formats, however, is that they can be reexecuted in an integrated computational environment that includes the supporting data files.

## Centering complex objects in scientific publishing with cloud infrastructure

Cloud infrastructure enables browser-based access to computational environments. A major challenge in extending these cloud infrastructures for scientific publishing is the associated cost, both for initial peer review as well as for the long-term preservation of included research objects. User-focused cloud technologies such as Binder (https://mybinder.org; [19]) enable easy access to these environments, but they do not directly address dataset storage. Neuroscience datasets may involve terabytes of data and hundreds of CPU hours of compute time, making cloud computing and data hosting nontrivial. Including multiple versions of a given dataset—from raw data to analysis-ready derivatives—only compounds this problem.

Creating economically viable, noncommercial options will likely involve the coordination of multiple academic and nonprofit groups such as the International Interactive Computing Collaboration (2i2c; https://2i2c.org) as well as explicit funding calls for projects advancing open standards through modular, composable infrastructure. Large field standard datasets, such as those provided by the Allen Institute for Brain Science (https://alleninstitute.org) or the International Brain Laboratory [20], are likely to further benefit from centralized data and computation. This approach has been pioneered in geosciences by the Pangeo project [21], which provides centralized access to and computation on field standard climatology data via JupyterHubs hosted on commercial clouds. Recently, Rokem and colleagues [22] have prototyped this approach in neuroscience through the development of a Pan-neuro initiative, encouraging optimism about future adoption in other scientific communities.

Smaller datasets collected by individual research groups, however, may require alternative approaches; in particular, decentralized data management offers a promising route forward to minimize reliance on a central hosting service in those cases where datasets are small enough to be duplicated [23]. NeuroLibre (https://neurolibre.com) provides one example of this model and relies on nonprofit support to host a curated collection of datasets, each of which support one or more NeuroLibre publications through hosted environments for reexecuting the described analyses.

**Fig 1:**
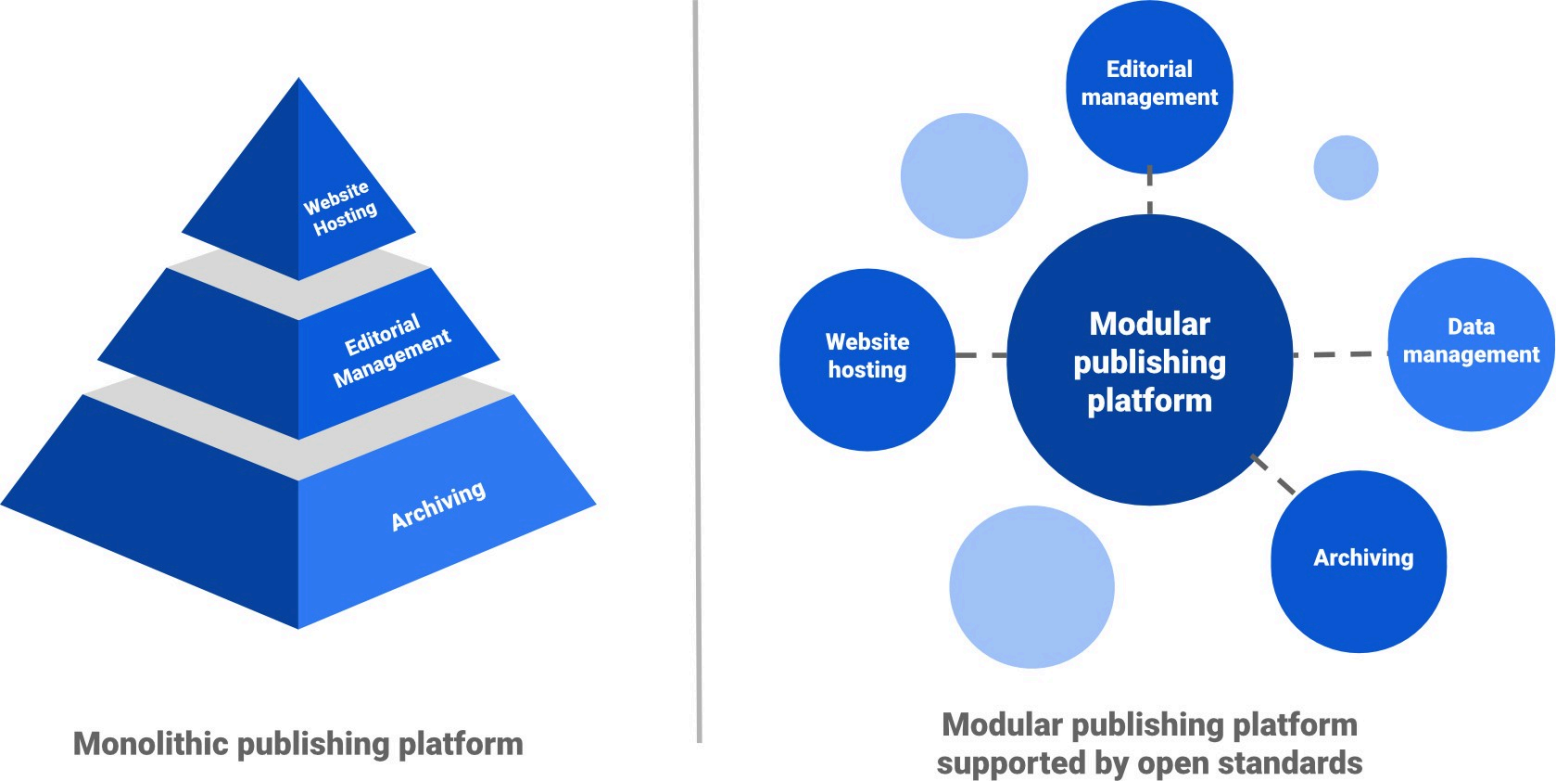
Contrasting monolithic and modular publishing platforms. While monolithic publishing platforms are self-contained, modular publishing platforms rely on open standards across composable infrastructure. In doing so, they create space for additional functionality such as data management that better supports scientific communities.

Although different in scale, we argue that both Pangeo and NeuroLibre share a core approach that should be more broadly adopted. By investing in infrastructure for integrated research objects that heavily relies on open, modular components, we can make strong contributions in individual research domains while still ensuring that these investments can be easily retooled and extended. Fig 1 contrasts this modular, composable infrastructure with more traditional publishing platforms developed on a monolithic technology stack.

NeuroLibre, for example, relies on a combined technology stack from the *Journal of Open Source Software* (JOSS; [24]), Jupyter Book, and BinderHub. Each of these projects independently combines modular technologies to meet existing community needs, and their combination—while currently unique to neuroscience—can easily be repurposed for other research communities, such as the development of Pan-neuro from the Pangeo model.

As scientists increasingly recognize the value in sharing their code and data [25], this approach could facilitate an important transition in scientific publishing. By leveraging MyST as an emerging standard for integrated research objects, alongside modular components for their hosting and reexecution through BinderHub and other open technologies, scientists will be better positioned to author articles that center all the research objects supporting a scientific result, in addition to the underlying narrative.

As science increasingly depends on digital infrastructure, it is clear that scientific publishing is at an inflection point. Reckoning with the Claerbout challenge will require providing access to the research objects supporting the actual scholarship rather than the “advertising” of static scientific articles. Adopting web-based technologies provides the strongest possible path forward, but managing this transition in the face of economic and sociological pressure requires academic communities to advocate for open and sustainable infrastructure development, as seen in the Pan-neuro and NeuroLibre initiatives. We argue that community-based efforts around open standards, modular and composable infrastructures, and new research object types will underpin the full potential of web-driven publishing.
